# Diagnostic Dilemma in Pediatric Subhepatic Appendicitis: A Report of Two Cases

**DOI:** 10.7759/cureus.87715

**Published:** 2025-07-11

**Authors:** Nusrath M P, Syeda Nousheen, Sarah Siddiqui, Asiya Mubeen, Lina Anwar

**Affiliations:** 1 Pediatric Emergency Medicine, Al Jalila Children's Specialty Hospital, Dubai, ARE; 2 Medicine, Dubai Medical University, Dubai, ARE; 3 Medical Education, Brunel Medical School, London, GBR

**Keywords:** acute appendicitis, appendectomy, laparoscopy, subhepatic appendix, ultrasonography

## Abstract

Subhepatic appendicitis is a rare anatomical variant of acute appendicitis that is caused by abnormal appendix positioning. It is an uncommon anatomical variation that poses diagnostic difficulties, especially in the pediatric population, where symptoms may be nonspecific. Its unusual presentation frequently results in a delayed diagnosis, increasing the possibility of complications like perforation and abscess formation. We report two pediatric cases of subhepatic appendicitis in previously healthy children aged 7 and 10 years who presented with abdominal pain and vomiting. Ultrasound aids in the diagnosis of these cases. In both of our cases, laparoscopic appendectomy was performed, and the recovery following the procedure was uneventful. These cases demonstrate how difficult it can be to diagnose subhepatic appendicitis in children because of its peculiar presentation, which can resemble gastric or hepatobiliary disorders. We report these cases with the objective of raising awareness among clinicians regarding the possibility of subhepatic appendicitis, particularly in patients with atypical upper abdominal pain, to highlight the signs and symptoms of this rare illness, and to underscore the value of ultrasonography as a diagnostic tool.

## Introduction

Acute appendicitis is the most common surgical emergency in children. While it usually presents with right lower quadrant pain, anatomical variations in the position of the appendix can lead to atypical clinical findings and diagnostic uncertainty. Although the appendix most commonly lies in a retrocecal position, other positions such as the subhepatic, pelvic, or intraherniary are also known to occur [1,2].

Subhepatic appendicitis, a rare variant, is typically due to intestinal malrotation or incomplete descent of the cecum during embryogenesis [3], characterized by the appendix's abnormal positioning under the liver. This reflects an uncommon anatomical variant where the appendix (and often the cecum) hasn't descended to the usual right lower quadrant during fetal development. In this location, the appendix may be present with pain in the right upper quadrant, which can mimic conditions like gastritis or cholecystitis. These atypical presentations often result in diagnostic delays and increase the risk of complications such as perforation or localized abscess formation.

Early diagnosis relies heavily on imaging, particularly abdominal ultrasound in children, though it is often limited in ectopic cases where the appendix is in an abnormal position. Dilated ileal loops in the right lower abdomen and a vermiform structure in the right upper abdomen may be seen on ultrasound when the appendix is in a subhepatic position, known as the fishbone sign. Surgical management is through a laparoscopic approach, as it helps with better visualization, faster recovery, and fewer postoperative complications [4]. 

Here, we describe two cases of subhepatic appendicitis diagnosed in previously healthy boys aged 7 and 10, respectively, who presented with abdominal pain and vomiting, and the diagnosis was made by ultrasound.

## Case presentation

Case 1

A seven-year-old previously healthy boy presented to the emergency department (ED) for the second time with severe abdominal pain and persistent vomiting. He had a fever for four days and a one-day history of abdominal pain, which started in the periumbilical region. He also experienced multiple episodes of non-bilious, non-projectile vomiting. The patient recently consumed food from a restaurant; however, no other individuals from the same gathering reported similar symptoms.

During his initial ED visit, he was diagnosed with acute gastritis and discharged after showing clinical improvement with symptomatic treatment. However, later that evening, his abdominal pain intensified and localized to the right side of the abdomen. The pain was described as severe, getting worse when standing or walking, and getting better when lying down. No bowel or urinary symptoms were present.

On examination, the patient was alert but appeared tired and nauseated. The patient was afebrile, and vitals were within normal limits. Temperature: 36.4°C, heart rate: 114/min (regular), respiration: 24/min (regular), blood pressure: 94/62 mmHg, oxygen saturation: 100% in room air. Abdominal examination revealed localized tenderness in the right lumbar and right hypochondrium with positive rebound tenderness. The rest of the abdomen was soft and non-distended. Rovsing’s sign was negative, and no hernias were present. Other systemic examinations were unremarkable.

Blood count, serum electrolytes, and renal function tests were within normal limits during the first visit. C-reactive protein was mildly high, 15.4 mg/l. In the second visit, the WBC count was above 10000, and it showed neutrophilia. C-reactive protein was increased to 32.2 mg/l, indicating an inflammatory or infectious process. Serum electrolytes and renal function tests were within normal limits (Table 1).

**Table 1:** Blood investigations MCV: Mean Corpuscular Volume; MCH: Mean Corpuscular hemoglobin; MCHC: Mean Corpuscular Hemoglobin Concentration; RDW: Red cell Distribution Width; MPV: Mean Platelet Volume, SGOT (AST): Aspartate aminotransferase, SGPT (ALT): Alanine aminotransferase

Component	Reference Range	First visit	Second visit
WBC count	5.0 - 13.0 10^3/uL	8.6	11.6
RBC count	4.00 - 5.20 10^6/uL	4.26	4.4
Hemoglobin	11.5 - 15.5 g/dL	11.3	11.8
Hematocrit	35.0 - 45.0 %	33.8	34.5
MCV	77.0 - 95.0 fL	79.3	78.4
MCH	25.0 - 29.0 pg	26.6	26.8
MCHC	31.5 - 34.5 g/dL	33.4	34.2
RDW	11.5 - 14.0 %	13.9	13.6
Platelets count	170 - 450 10^3/uL	261	286
MPV	7.4 - 10.4 fL	8.5	8.6
Neutrophil Absolute	2.0 - 8.0 10^3/uL	6.54	8.25
Lymphocytes Absolute	1.00 - 5.00 10^3/uL	1.51	2.18
Monocytes Absolute	0.20 - 1.00 10^3/uL	0.56	1.13
Eosinophils Absolute	0.10 - 1.00 10^3/uL	0	0
Basophils Absolute	0.00 - 0.10 10^3/uL	0.02	0.02
Neutrophil %		75.8	71.2
Lymphocyte %		17.5	18.8
Monocyte %		6.5	9.8
Eosinophil %		0	0
Basophil %		0.2	0.2
Sodium		135	138
Potassium	3.5 - 5.1 mmol/L	3.3	3.8
Chloride	97 - 107 mmol/L	107	107
Bicarbonate (HCO3)	17 - 27 mmol/L	16	18
Creatinine	0.52 - 0.69 mg/dL	0.38	0.37
Urea	19.26 - 47.294 mg/dL	19	14
Calcium	8.8 - 10.8 mg/dL	9	8.9
Glucose, Random	73 - 112 mg/dL	114	100
SGOT(AST)	0 - 51 U/L		26
SGPT(ALT)	0 - 39 U/L		41
Alkaline Phosphatase	153 - 367 U/L		150
Total Protein	6.4 - 7.7 g/dL		7.1
Bilirubin, Total	0 - 1.2 mg/dL		0.25
Albumin	3.8 - 5.4 g/dL		4.3
C-reactive protein	0 - 5 mg/L	15.4	32.2
Procalcitonin (PCT)	< 0.5 ng/mL	0.05	

**Figure 1:**
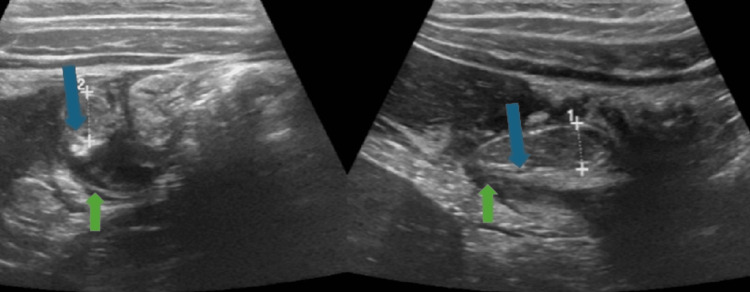
Ultrasound showing subhepatic appendix measuring 8.4 mm in diameter and minimal free fluid in the subhepatic region Blue arrow: Subhepatic appendix; Green arrow: free minimal fluid in the subhepatic region.

An abdominal ultrasound revealed a subhepatic appendix measuring 8.4 mm in diameter, with surrounding echogenic fat, adjacent enlarged mesenteric lymph nodes, and free minimal fluid in the subhepatic region (Figure 1).

The patient underwent laparoscopic appendectomy under general anesthesia. The appendix was found to be in a subhepatic position, with the tip perforated. Analgesics and antibiotics were administered intravenously to the patient. Following a smooth recovery, the patient was discharged on the eighth day in good health with the appropriate follow-up instructions.

Case 2

A 10-year-old previously healthy boy presented to the emergency department (ED) with moderate to severe abdominal pain localized to both the right and left quadrants. The pain was rated 8 out of 10 in intensity, exacerbated by positional changes, and non-radiating. It was associated with episodes of vomiting, fever, and a recent history of cough. The patient also reported not passing stool for the past two days but denied any urinary symptoms. This patient was seen in the ED the previous day with fever, cough, vomiting, and left upper abdominal pain. A chest X-ray showed patchy consolidation in the right upper lung zone, diagnosed as pneumonia, and the patient was discharged on antibiotics.

On examinations, the patient appeared uncomfortable and restless. The patient was afebrile, and vitals were within normal limits. Temperature: 36.8°C, heart rate: 92/min (regular), respiration: 22/min (regular), blood pressure: 115/79 mmHg, oxygen saturation: 99% in room air. Abdominal examination showed tenderness and guarding in the right lumbar, right and left lower quadrants, and positive rebound tenderness. Other systemic examinations were unremarkable.

Laboratory investigations revealed leukocytosis, a WBC count above 10000, and neutrophilia. C-reactive protein level was elevated to 14.9 mg/L, indicating an inflammatory or infectious process. Other blood parameters, liver function tests, serum electrolytes, and renal function tests were within normal limits (Table 2).

**Table 2:** Blood investigations MCV: Mean Corpuscular Volume; MCH: Mean Corpuscular Hemoglobin; MCHC: Mean Corpuscular Hemoglobin Concentration; RDW: Red Cell Distribution Width; MPV: Mean Platelet Volume, SGOT (AST): Aspartate aminotransferase, SGPT (ALT): Alanine aminotransferase

Component	Reference Range	First visit	Second visit
WBC count	5.0 - 13.0 10^3/uL	13.9	20.7
RBC count	4.00 - 5.20 10^6/uL	5.35	5.04
Hemoglobin	11.5 - 15.5 g/dL	15.1	14.3
Hematocrit	35.0 - 45.0 %	42	40.2
MCV	77.0 - 95.0 fL	78.5	79.8
MCH	25.0 - 29.0 pg	28.2	28.4
MCHC	31.5 - 34.5 g/dL	36	35.6
RDW	11.5 - 14.0 %	12.4	12.4
Platelets count	170 - 450 10^3/uL	331	367
MPV	7.4 - 10.4 fL	9.4	9.4
Neutrophil absolute	2.0 - 8.0 10^3/uL	12.66	18.33
Lymphocytes absolute	1.00 - 5.00 10^3/uL	0.85	0.97
Monocytes absolute	0.20 - 1.00 10^3/uL	0.3	1.31
Eosinophils absolute	0.10 - 1.00 10^3/uL	0.04	0.05
Basophils absolute	0.00 - 0.10 10^3/uL	0.05	0.04
Neutrophil %		91	88.6
Lymphocyte %		6.1	4.7
Monocyte %		2.2	6.3
Eosinophil %		0.3	0.2
Basophil %		0.4	0.2
Sodium		134	138
Potassium	3.5 - 5.1 mmol/L	4	4
Chloride	97 - 107 mmol/L	99	100
Bicarbonate (HCO3)	17 - 27 mmol/L	22	20
Creatinine	0.52 - 0.69 mg/dL	0.48	0.5
Urea	19.26 - 47.294 mg/dL	24	40
Calcium	8.8 - 10.8 mg/dL	9.2	9.9
Glucose, Random	73 - 112 mg/dL	105	125
SGOT (AST)	0 - 51 U/L	30	36
SGPT (ALT)	0 - 39 U/L	12	13
Alkaline Phosphatase	153 - 367 U/L	158	173
Total Protein	6.4 - 7.7 g/dL	7.1	8
Bilirubin, Total	0 - 1.2 mg/dL	0.33	0.32
Albumin	3.8 - 5.4 g/dL	4.1	4.7
C-reactive protein	0 - 5 mg/L	15.4	14.9
Procalcitonin (PCT)	< 0.5 ng/mL	0.05	0.17

**Figure 2:**
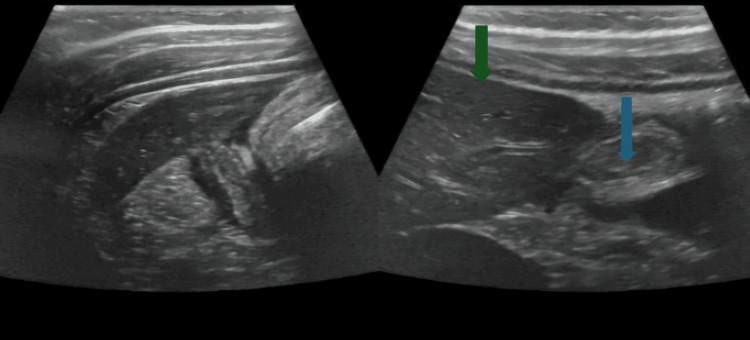
Ultrasound showing subhepatic appendix Blue arrow: Subhepatic appendix, Green arrow: Liver

Abdominal ultrasonography demonstrated features of acute appendicitis with the appendix located in the subhepatic region. The appendix appeared distended, measuring 9.2 mm in diameter, with surrounding inflammatory changes and tenderness on probe pressure (Figure 2).

The patient underwent laparoscopic appendectomy under general anesthesia. The appendix was found to be inflamed with localized peritonitis and gangrene without perforation or abscess. Analgesics and antibiotics were administered intravenously to the patient. The patient made an uneventful recovery and was discharged on the sixth day in satisfactory condition and with appropriate follow-up instructions.

## Discussion

In children, acute appendicitis is the most common cause of abdominal surgical emergencies. While the classic presentation includes right lower quadrant pain, fever, nausea, and anorexia, anatomical variations in the position of the appendix can obscure this clinical picture [5]. One such uncommon variant is subhepatic appendicitis, where the appendix lies in the subhepatic space due to either a congenital malrotation or abnormal cecal fixation.

When presenting with acute symptoms of inflammation, the location of the appendix may present a diagnostic challenge [3]. In 65% of cases, the appendix is found in the retrocecal position; in decreasing order of frequency, it is less frequently found in the pelvis, subcecal, pre-, and post-ileal locations. The other less common positions include subhepatic, lateral pouch, mesocoeliac, left-sided, and intraherniary [6]. King was the first to report subhepatic appendix in 1955 [3,7]. Subhepatic appendicitis is very rare; according to a study by Palanivelu et al., only 0.08% of 7210 patients had subhepatic appendicitis, and most of those cases were retrocecal [8].

Subhepatic appendicitis presents a diagnostic challenge, especially in pediatric patients. The pain may localize to the right upper quadrant or appear more diffuse, often mimicking hepatobiliary, gastric, or renal pathologies. In both cases we encountered, the atypical location of the appendix contributed to either an initial misdiagnosis or a delayed diagnosis. This aligns with existing literature, which suggests that subhepatic appendicitis is often mistaken for conditions such as cholecystitis, gastritis, or even pneumonia due to overlapping symptoms [9]. In one of our cases, the initial presentation was misdiagnosed as acute gastritis. The other case was diagnosed as pneumonia.

Although an abdominal ultrasound scan is the initial radiological test, there is a significant chance that it will be misdiagnosed [9]. Abdominal sonography has poor sensitivity for diagnosing unusually located appendicitis. However, because a computed tomography (CT) scan has been shown to have high sensitivity (100%), specificity (95%), and accuracy (98%) in diagnosing acute appendicitis [10], it is the most effective modality for identifying subhepatic appendicitis [6]. Imaging plays a crucial role in diagnosis, particularly ultrasonography and CT, though the latter is less frequently used in children due to radiation exposure concerns. Even though abdominal ultrasonography is the most widely used diagnostic method, CT scans offer a high level of diagnostic sensitivity. Laparoscopy is frequently used to make a diagnosis, especially in cases where a CT scan is unclear. In our cases, ultrasound successfully identified the subhepatic location of the inflamed appendix, allowing timely surgical intervention. Imaging is crucial for early diagnosis, especially abdominal ultrasonography in children, to prevent complications and delays in diagnosis. In one of our cases, perforation had already occurred by the time of surgery, highlighting the risks associated with delayed diagnosis.

Surgically, laparoscopic appendectomy remains the standard of care, even in anatomically atypical cases. However, the unusual position may increase the technical difficulty of the procedure, necessitating careful port placement and cautious dissection. Laparoscopic surgery is used for surgical management because it is a safe procedure, improves visualization, speeds up recovery, and reduces postoperative complications [4,11]. Our cases were successfully treated with a laparoscopic appendectomy and had an uneventful recovery without complications.

While subhepatic appendicitis is more commonly reported in adults [12], pediatric cases are rare and underrepresented in the literature. This underlines the importance of maintaining a high index of suspicion for anatomical variants in children presenting with atypical abdominal pain, particularly when initial investigations are inconclusive.

## Conclusions

A subhepatic appendix is an unusual condition caused by either the non-descent of the cecum or intestinal malrotation in the early stages of development. When inflamed, this can cause misdiagnosis because it can mimic other pathologies in organs that are normally found there, resulting in perforation and abscess formation, which can raise morbidity and potentially mortality.

Subhepatic appendicitis, although rare, should be considered in children presenting with atypical upper abdominal pain and systemic signs of inflammation. A high index of suspicion and appropriate imaging are essential for timely diagnosis and management. Prompt imaging and surgical exploration are crucial to avoid complications like perforation. Laparoscopic appendectomy remains the gold standard for diagnosis and treatment in such anatomically variant cases.
